# Comparable results between crosslinked polyethylene and conventional ultra-high molecular weight polyethylene implanted in total knee arthroplasty: systematic review and meta-analysis of randomised clinical trials

**DOI:** 10.1007/s00167-022-06879-7

**Published:** 2022-02-19

**Authors:** Alessandro Bistolfi, Fortunato Giustra, Francesco Bosco, Carlotta Faccenda, Marianna Viotto, Luigi Sabatini, Paola Berchialla, Veronica Sciannameo, Eugenio Graziano, Alessandro Massè

**Affiliations:** 1 Orthopaedics and Traumatology, Ospedale Cardinal Massaia Asti, via Conte Verde 125, 14100 Asti, Italy; 2 University of the Studies of Turin, Turin, Italy; 3 AO Città della Salute e della Scienza, Turin, Italy; 4 Department of Clinical and Biological Sciences, University of Torino, Turin, Italy

**Keywords:** Polyethylene, CPE, UHMWPE, Crosslinked, Knee, Total knee arthroplasty, Meta-analysis

## Abstract

**Purpose:**

Total knee arthroplasty (TKA) has experienced exponential growth over the last decade, including increasingly younger patients with high functional demands. Highly crosslinked polyethylene (HXLPE) has been proven effective in reducing osteolysis and loosening revisions while improving long-term survival and performance in total hip arthroplasty; nevertheless, this superiority is not demonstrated in TKA. The aim of this systematic review and meta-analysis was to examine whether HXLPE improved overall survival and postoperative functional and radiological outcomes compared to conventional polyethylene (CPE) in TKA.

**Methods:**

According to the Preferred Reporting Items for Systematic Reviews and Meta-Analyses (PRISMA) guideline, a literature search of five databases (PubMed, Medline, Scopus, Science Direct and Embase) was made. A PICOS model was performed. The initial screening identified 2541 studies. Each eligible clinical article was analysed according to the Oxford Centre for Evidence-Based Medicine 2011 Levels of Evidence (LoE). Only randomised clinical trials (RCTs) of LoE 1 and 2 were included. The methodological quality of the articles was assessed using the Risk of Bias 2 (RoB 2) tool.

**Results:**

Six clinical studies were included in the final study. This systematic review and meta-analysis were registered on the International Prospective Register of Systematic Reviews (PROSPERO). A total of 2285 knees were included. Eight outcomes (total reoperations, reoperations for prosthesis loosening and infections, radiolucent lines, osteolysis, mechanical failure, postoperative KSS knee score and function score) were analysed. For none of them, a statistically significant difference was found about the superiority of HXLPE over CPE (*p* > 0.05).

**Conclusions:**

There were no statistically significant differences between HXLPE and CPE for TKA concerning clinical, radiological, and functional outcomes; nevertheless, HXLPE did not show higher failure rates or complications and can be safely used for TKA.

**Level of evidence:**

II.

## Introduction

Total knee arthroplasty (TKA) is the most common joint arthroplasty in North America and the second most common in Europe [35]. Therefore, this situation has led to new materials to improve the performance and durability of prosthetic implants.

One of the main factors affecting the long-term survival of a TKA is polyethylene wear-related osteolysis as a cause of aseptic loosening [3, 46, 52]. Furthermore, the particle sizes of polyethylene have been correlated to their biological activity; in particular, smaller particle sizes exhibited greater reactivity [14, 16, 19, 21].

As a result of the success of THA, great interest has been expressed in applying highly crosslinked polyethylene (HXLPE) to TKA [4, 8, 18, 37, 48]. Promising results supporting HXLPE in TKA have emerged from in vitro studies [43, 44]; the same benefits have not yet been confirmed in in vivo studies [16, 20, 49]. Several clinical studies have reported conflicting results regarding the superiority of HXLPE over CPE in TKA [17, 24, 39]. In addition, knee and hip joints present different tribological and kinematic characteristics. Polyethylene wear is more significant in TKA than in THA, while on the contrary, mechanical stresses are higher on tibial liners than on acetabular cups [5, 8, 28]. Therefore, since the crosslinking process of polyethylene determines a reduction of the mechanical properties, it may also increase the risk of tibial insert fracture [36, 40].

The aim of our systematic review and meta-analysis of randomised clinical trials (RCTs) was to evaluate whether HXLPE improved overall survivorship and postoperative functional and radiological outcomes compared with CPE in TKA since its introduction or, on the contrary, if HXLPE determined a higher risk of revisions than CPE.

## Methods

### Research question

The Preferred Reporting Items for Systematic Reviews and Meta-Analyses (PRISMA) checklist was used to perform this systematic review and meta-analysis [33]. The possible clinical and radiological improvements have been researched using HXLPE over CPE in TKA. Four authors (FB, CF, FG and MV) searched and evaluated the articles independently to avoid possible bias. In discrepancies, a fifth author (AB) was consulted to resolve any additional uncertainties. The Patient, Intervention, Comparison, Outcomes, and Study (PICOS) design was used to frame and answer clinical questions according to the PRISMA checklist [33]: patient (P), patients who had undergone primary TKA; intervention (I), HXLPE tibial insert in TKA; comparison (C), CPE tibial insert in TKA; outcomes (O), clinical, functional, and radiographic characteristics of the postoperative results of HXLPE compared with CPE in TKA; Study design model (S), RCTs.

### Inclusion criteria

The inclusion criteria of the studies examined were “articles published in the English language, studies published within the last 15 years and with a minimum follow-up of 1 year, only RCTs with LoE 1 and 2, the full-text of the articles were available, and participants underwent primary TKA using HXLPE or CPE”. “Biochemical and in vitro studies, case reports, editorials, book chapters, technical reports, preclinical studies, and review articles” were excluded from the research. Studies about human subjects were exclusively considered.

### Search strategy and study screening

**Fig. 1:**
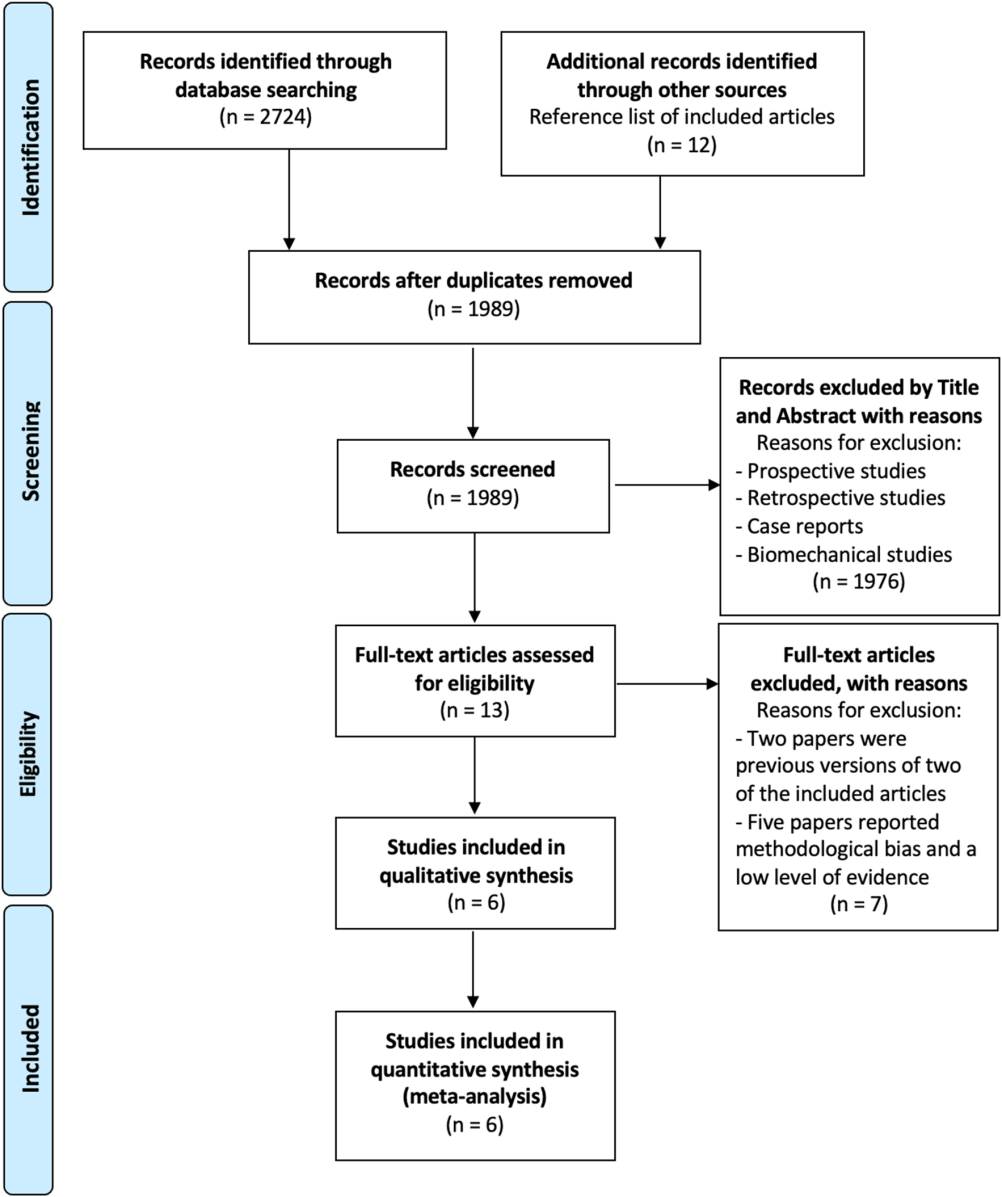
Preferred Reporting Items for Systematic Reviews and Meta-Analyses (PRISMA) flow diagram of articles screened, selected, and included in the systematic review and meta-analysis

Literature research in five databases (PubMed, Medline, Scopus, Science Direct, and Embase) was performed using the following MeSH terms: [(knee replacement) OR (knee arthroplast*) OR (knee revision) OR (TKA) OR (TKR)) AND ((polyethylene) OR (crosslink) OR (CPE) OR (UHMWPE) OR (HXLPE)]. The research was limited from January 2005 to September 2021. A total of 2724 studies were identified through the database searches. After exclusion of duplicates, 1989 studies were included, of these, 1976 were excluded after examining the title and abstract. After the full-text evaluation for eligibility of these 13 studies, according to the inclusion and exclusion criteria, 6 clinical studies [16, 23, 25, 26, 29, 42] that evaluated differences in clinical and radiological outcomes using HXLPE versus CPE in TKA were included in the analysis. The bibliography for each article was reviewed to find additional relevant publications. The PRISMA flow chart for reporting study selection is shown in Fig. 1.

### Quality assessment

**Fig. 2:**
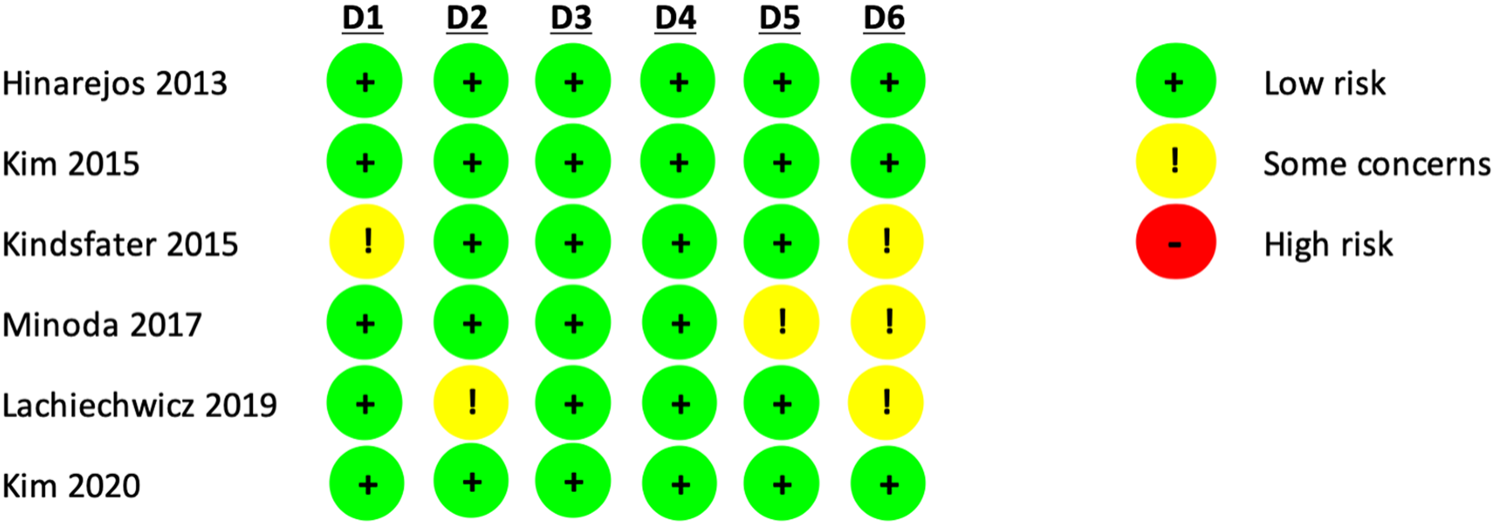
Risk of bias conformed by the Cochrane Handbook for Systematic Reviews of Interventions. The quality and risk of bias of individual randomised controlled trials included in the meta-analysis. *D1* randomisation process, *D2* Deviations from the intended interventions, *D3* missing outcomes data, *D4* measurement of the outcome, *D5* Selection of the reported result, *D6* overall

According to the Oxford Centre for Evidence-Based Medicine 2011 Levels of Evidence (LoE) [9], each clinical article was analysed. The LoE is an effective tool for assessing the strength of findings in research studies. Articles were graded from 1 to 5, where LoE 1 and 2 mean better study design, methodological quality, and lower risk of bias. The methodological quality of the articles was evaluated through the Risk of Bias 2 (RoB 2) tool [54] by four authors (Fig. 2). A fifth author resolved any cases of disagreement. The statistical analysis was performed by professional statisticians (PB and VS). All authors participated equally in the study design, manuscript preparation, and final review. This systematic review and meta-analysis were registered on the International Prospective Register of Systematic Reviews (PROSPERO), CRD42021231100 in March 2021 [53].

### Data extraction

Data from the selected studies were inserted in a standard template and included the following study characteristics: author and publication year, number of knees, sample size mean age, sample size percentage sex, body mass index (BMI), follow-up of the studies, study design, total reoperations, reoperations for prosthesis loosening, reoperations for infections, radiolucent lines, osteolysis, mechanical failure related to the tibial polyethylene, and postoperative Knee Society Score (KSS) knee score and function score (Tables 1 and 2).

**Table 1:** Main demographic characteristics of patients collected in randomised clinical trials (RCTs) included in the systematic review and meta-analysis

Author and publication year	Sample size patients, no		Numbers of knees, no		M, no (%)		F, no (%)		Age y.o., mean ± SD		BMI, mean ± SD		Follow-up months	
	HXLPE	CPE	HXLPE	CPE	HXLPE	CPE	HXLPE	CPE	HXLPE	CPE	HXLPE	CPE	HXLPE	CPE
Hinarejos et al., 2013 [16]	14	14	14	14	3 (23.5)	3 (23.5)	11 (76.5)	11 (76.5)	71.6 ± 7.6	74.2 ± 5.1	30.9 ± 3.9	30.5 ± 6.5	12	12
Kim et al. 2015, [23]	177	177	177	177	27 (15.2)	27 (15.2)	150 (84.8)	150 (84.8)	58 ± 8	58 ± 8	26	26	72	72
Kindsfater et al., 2015 [26]	477	449	477	449	169 (35.4)	159 (35.4)	308 (64.6)	290 (64.6)	66.4 ± 8.5	66.3 ± 8.5	32.6 ± 6.8	33.2 ± 7.3	60	60
Minoda et al., 2017 [42]	8	8	8	8	8 (100)	8 (100)	0 (0)	0 (0)	77.6 ± 5.9	73.5 ± 11.7	26 ± 3.2	26.9 ± 2.3	12	12
Lachiewicz et al., 2019 [29]	152	145	162	155	48 (31.7)	45 (31.2)	104 (68.3)	100 (68.8)	68.1 ± 10	69.7 ± 9.5	31.5 ± 5.4	31.4 ± 5.9	68	70.2
Kim et al., 2020 [25]	319	319	319	319	104 (32.6)	104 (32.6)	215 (67.4)	215 (67.4)	60.3 ± 4.3	60.3 ± 4.3	29.1 ± 6.1	29.1 ± 6.1	158.4	158.4
Total, No. (%)	1147	1112	1157	1122	359 (31.3)	346 (31.1)	788 (68.7)	766 (68.9)						

*M* male, *F* female, *BMI* body mass index, *No* number of evaluation cases, *%* percentage, *y.o*. years old, *SD* standard deviation, % percentage, *HXLPE* highly crosslinked polyethylene, *CPE* conventional polyethylene

**Table 2:** Clinical and radiological outcomes in total knee arthroplasty (TKA) comparing highly crosslinked polyethylene (HXLPE) to conventional polyethylene (CPE)

Author and publication year	Study design	Total reoperations, no (%)		Reoperations for prosthesis loosening, No (%)		Reoperations for infections, no. (%)		Radiolucent lines, no (%)		Osteolysis, no (%)		Mechanical failures related to the tibal polyethylene, no (%)		Post-operative KSS knee score Mean ± SD		Post-operative KSS function score Mean ± SD	
		HXLPE	CPE	HXLPE	CPE	HXLPE	CPE	HXLPE	CPE	HXLPE	CPE	HXLPE	CPE	HXLPE	CPE	HXLPE	CPE
Hinarejos et al., 2013 [16]	RCT													92.1 ± 4.6	93.5 ± 2.8	85.9 ± 15.7	77.6 ± 13.9
Kim et al. 2015, [23]	RCT	1 (0.6)	1 (0.6)	0 (0)	0 (0)			0 (0)	0 (0)	0 (0)	0 (0)	0 (0)	0 (0)				
Kindsfater et al., 2015 [26]	RCT	6 (1.3)	10 (2.2)	1 (0.2)	3 (0.7)	1 (0.2)	4 (0.9)	13 (2.7)	14 (3.1)	2 (0.4)	3 (0.7)			93.1 ± 8.5	93.5 ± 9.2	82.9 ± 21.6	80.4 ± 23.1
Minoda et al., 2017 [42]	RCT													96 ± 9	96 ± 7	78 ± 25	79 ± 16
Lachiewicz et al., 2019 [29]	RCT	3 (1.9)	6 (3.9)	0 (0)	1 (0.6)	3 (1.9)	3 (1.9)	22 (13.6)	21 (13.5)	0 (0)	4 (2.6)	0 (0)	0 (0)	91.9 ± 8.3	91.7 ± 9.4	66.5 ± 31.1	65.2 ± 28.7
Kim et al., 2020 [25]	RCT	6 (1.9)	7 (2.2)			3 (0.9)	4 (1.3)			0 (0)	0 (0)	0 (0)	0 (0)	93 ± 5	92 ± 6	86	86

*KSS* Knee Society Score, *No* number of evaluation cases, *%* percentage, *SD* standard deviation, *RCT* randomised clinical trial, *HXLPE* highly crosslinked polyethylene, *CPE* conventional polyethylene

### Data analysis

The DerSimonian and Laird random-effects model was used to pool estimates across studies. Average effect size and a 95% confidence interval (CI) was computed by the Jackson method. To estimate heterogeneity between studies, Cochran’s Q test and Higgins’ *I*^2^ statistic were used. Values of *I*^2^ of 0–24.9%, 25–49.9%, 50–74.9%, and > 75% suggested no, low, moderate, or high heterogeneity, respectively. The pooled incidence rate ratio (IRR) and the pooled standardised mean difference (SMD) were considered statistically significant with a *p* value < 0.05. Finally, publication bias was visually inspected by funnel plots and tested by Egger's test. Statistical analyses were performed with R software, version 4.0.5 (2020; R Core Team, Vienna, Austria).

## Results

A total of 2285 knees were analysed during a mean follow-up of 63.7 ± 53.7 months. The main demographic characteristics, such as age, percentage of males and females, and BMI, are reported in Table 1.

**Fig. 3:**
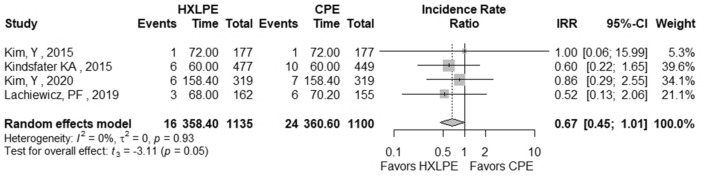
Total reoperations. *HXLPE* highly crosslinked polyethylene, *CPE* conventional polyethylene, *IRR* incidence rate ratio, *CI* confidence interval, *p* *p* value

**Fig. 4:**
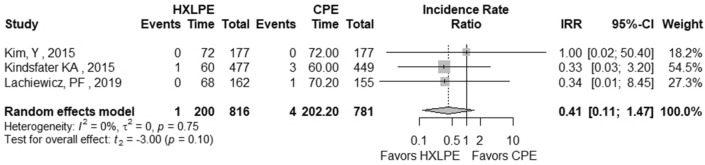
Reoperations for prosthesis loosening. *HXLPE* highly crosslinked polyethylene, *CPE* conventional polyethylene, *IRR* incidence rate ratio, *CI* confidence interval, *p* *p* value

**Fig. 5:**
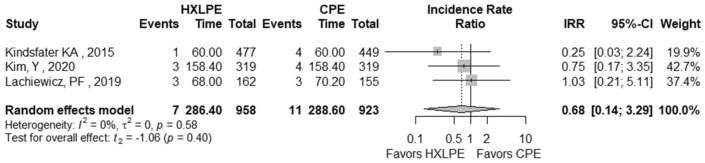
Reoperations for infections. *HXLPE* highly crosslinked polyethylene, *CPE* conventional polyethylene, *IRR* incidence rate ratio, *CI* confidence interval, *p* *p* value

**Fig. 6:**
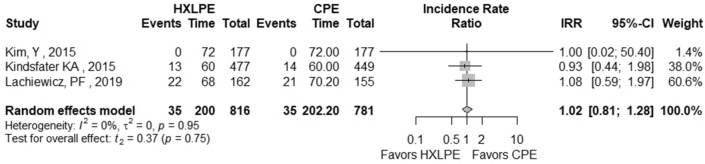
Radiolucent lines. *HXLPE* highly crosslinked polyethylene, *CPE* conventional polyethylene, *IRR* incidence rate ratio, *CI* confidence interval, *p* *p* value

**Fig. 7:**
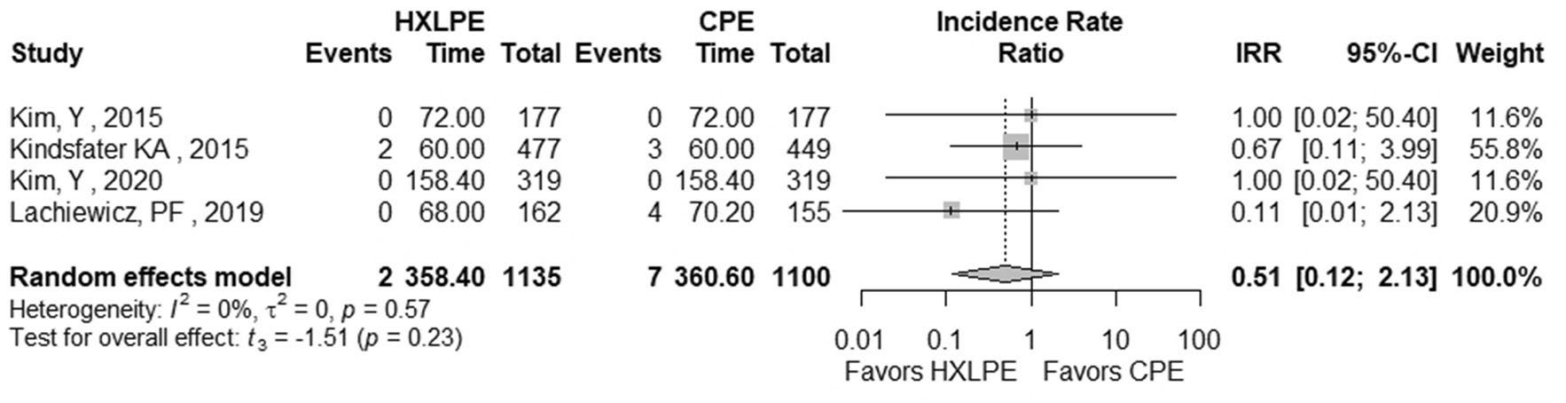
Osteolysis. *HXLPE* highly crosslinked polyethylene, *CPE* conventional polyethylene, *IRR* incidence rate ratio, *CI* confidence interval, *p* *p* value

**Fig. 8:**
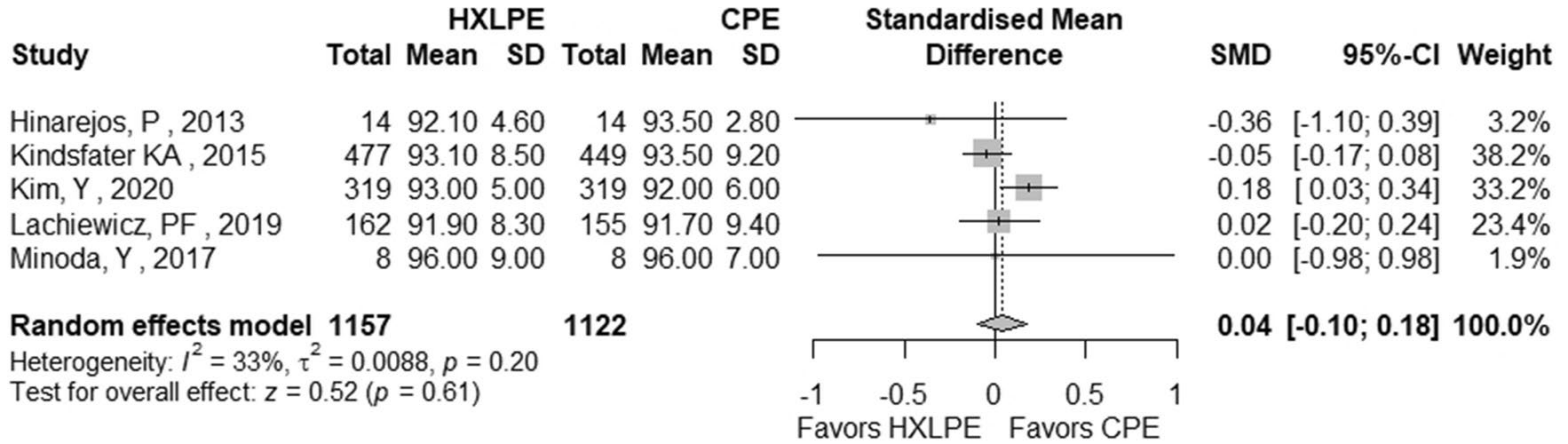
Postoperative KSS knee score. *KSS* Knee Society Score, *HXLPE* highly crosslinked polyethylene, *CPE* Conventional Polyethylene, *SMD* standardised mean difference, *CI* confidence interval, *p* *p* value

**Fig. 9:**
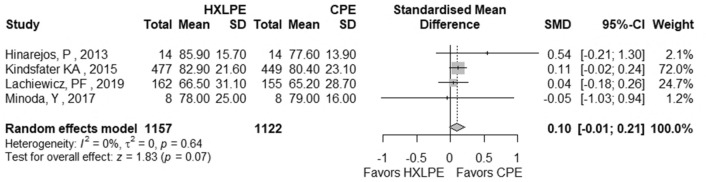
Postoperative KSS function score. *KSS* Knee Society Score, *HXLPE* highly crosslinked polyethylene, *CPE* Conventional Polyethylene, *SMD* standardised mean difference, *CI* confidence interval, *p* *p* value

The outcomes total reoperations, reoperations for prosthesis loosening, reoperations for infections, radiolucent lines, osteolysis, mechanical failures, and postoperative KSS knee score and function score were examined (Table 2). A meta-analysis was performed for seven of these parameters, while it was not possible for the outcome of “mechanical failure” because none of the studies reported mechanical failures specifically related to the tibial polyethylene. For each outcome, the analysis did not show any significant publication bias effect. No significant heterogeneity was observed in the statistical analysis results for each outcome analysed in the included studies [16, 23, 25, 26, 29, 42]. There was no statistically significant difference found regarding the superiority of HXLPE over CPE for all outcomes assessed above (*p* > 0.05) (Figs. 3, 4, 5, 6, 7, 8, 9).

## Discussion

The most important finding of the present study was a tendency towards the clinical and radiographic superiority of HXLPE over CPE, although it was not statistically significant for all outcomes. This finding could be relevant from a clinical point of view, because it may allow the safe use of crosslinked polyethylene for TKA.

According to the literature, the main cause of early revision is a prosthetic infection, whereas the main cause of late revision is aseptic loosening [10, 11, 27]. Revision total knee arthroplasty (RTKA) is a highly demanding procedure, with both high complication and postoperative failure rates [58]. Therefore, it is crucial to consider every prognostic factor that could predict a higher or lower risk of revision, as in this specific case, the performance and durability of the tibial polyethylene liner.

Regarding aseptic loosening, it is well known that the longevity of the implant is closely related to the wear of the polyethylene component, which leads to debris and subsequently to aseptic loosening with the need for revision [6, 12]. It is also essential to adopt all strategies that could reduce the infection rate. A recent analysis examining the risk of infection with alternative bearing surfaces in TKA reported that HXLPE had a 26% lower revision risk for infection than CPE [56]. Furthermore, the capability of the polyethylene substrate to avoid bacterial adhesion and biofilm formation could also play a role in infection risk [2]. It has been demonstrated that HXLPE has potentially greater resistance to bacterial adhesion and biofilm formation than CPE [1]. For these reasons, HXLPE has been adopted in TKA. In a recent meta-analysis, which considered national registries, Gkiatas et al. showed that patients in whom HXLPE had been implanted were less likely to be revised following aseptic loosening than those in whom CPE had been implanted. Regarding the overall revision rate, no significant difference was found between the two types of implanted polyethylene [15]. However, our quantitative analysis showed a lower revision rate in the HXLPE group compared to that in the CPE group for infection and aseptic loosening, although this finding was statistically insignificant.

Other issues regard periprosthetic osteolysis, with rates after TKA ranging from 5 to 20% over a follow-up period of 5–15 years, and radiolucent lines [30, 31, 46]. In their meta-analyses, Yu et al. and Gkiatas et al. [15, 57] reported a low incidence of osteolysis comparing HXLPE with CPE. The authors supposed that this difference could be related to the shorter follow-up period (2–6 years) of the studies included in their meta-analysis. Our results found that this low incidence of osteolysis among the two groups was unchanged over the long-term follow-up (60–158.4 months).

From a biochemical perspective, HXLPE is more resistant than CPE to adhesive and abrasive wear, although it is associated with weaker mechanical properties, including lower toughness, ductility, and fatigue fracture resistance [7, 47, 50]. The superior performance, in terms of wear resistance, of HXLPE in THA has led to its use in TKA. On the contrary, previous studies [22, 32, 36, 51] have shown that HXLPE could lead to mechanical failure in TKA, and one of the reasons attributed to the failure of HXLPE in TKA is that wear mechanisms in the knee are not the same as those in the hip [5, 24]. Nevertheless, in favour of HXLPE, Yu et al. and Gkiatas et al. [15, 57], in their meta-analyses, found no mechanical failures for both polyethylene groups, as well as in the results of the studies included in our research where, again, no mechanical failures were reported [23, 25, 29]. Therefore, HXLPE appears to be as safe as CPE in TKA.

In the literature, contradictory evidence is reported about the superiority of HXLPE over CPE in TKA regarding clinical and functional results [38, 41]. Between the different validated clinical and functional scores, the most widely used is the KSS, consisting of two sections: the knee score and function score [34]. The data analysis revealed almost comparable results between the two types of polyethylene in the KSS knee score, although HXLPE tended to be superior to CPE in the KSS function score, despite being statistically insignificant. Other clinical and functional scores [34] were also described between the studies included, but it was not possible to perform a quantitative analysis. Kindsfater et al. [26] observed similar Western Ontario and McMaster Universities Osteoarthritis Index (WOMAC) [13] values in the postoperative period between the two types of liners compared. Minoda et al. [42] did not find a benefit in the use of HXLPE over CPE in either range of motion (ROM) or University of California Los Angeles (UCLA) activity score [45] in the postoperative period. Lachiewicz et al. [29], in their study, found similar results comparing the lower extremity activity score (LEAS) [55] in the two polyethylene groups. Kim et al. [25], although they reported better postoperative outcomes in the HXLPE group, also find statistically insignificant differences in the WOMAC, ROM, UCLA activity score and patient satisfaction.

Regardless of the score used, no clear postoperative clinical and functional superiority of HXLPE over CPE was found in the studies analysed. In our opinion, the clinical performance is more strictly related to other factors, such as implant design, alignment, and surgical technique, than to the material itself, which benefits may be evident in other aspects (such as reduced wear, mechanical failures and fractures and more extended durability).

This meta-analysis presents limitations that need to be considered. These were mainly related to the limited number of included studies; Minoda et al. [42] and Hinarejos et al. [16] had small sample sizes that could provide a lower statistical analysis. Additional clinical studies with larger samples of patients will be necessary to further evaluate the superiority of HXLPE over CPE in TKA. Furthermore, two designs produced by different brands were implanted in the studies analysed: cruciate-retaining (CR) and posterior stabilised (PS). Both designs lead to different kinematics in TKA. These could result in different forces being applied to the polyethylene liner and consequently may produce different wear. Moreover, a quantitative analysis was not possible for one of the outcomes studied, mechanical failure, because it was not observed in any of the studies examined. Finally, a wide variety of follow-ups with a range of 12–158.4 months was reported in the studies included in this meta-analysis. A more homogeneous clinical follow-up would improve the validity of the data.

Previous studies [15, 57] and our meta-analysis have not shown a statistically significant superiority of HXLPE over CPE, although this paper has shown a tendency of the superiority of HXLPE over CPE. Further studies would be helpful to corroborate these findings to improve TKA outcomes.

## Conclusions

This systematic review and meta-analysis showed statistically insignificant differences between HXLPE and CPE for TKA regarding clinical, radiological, and functional outcomes. The superiority of HXLPE over CPE remains unproven; nevertheless, it did not show higher rates of failure or complications with respect to the standard material and, considered the superiority demonstrated in laboratory-studies, it could be used for TKA.

## Abbreviations

UHMWPE: Ultra-high molecular weight polyethylene
TKA: Total knee arthroplasty
HXLPE: Highly crosslinked polyethylene
CPE: Conventional polyethylene
PRISMA: Preferred Reporting Items for Systematic Reviews and Meta-Analyses
LoE: Oxford Centre for Evidence-Based Medicine 2011 Levels of Evidence
RCTs: Randomised clinical trials
RoB 2: Risk of Bias 2
PROSPERO: International Prospective Register of Systematic Review
MPS: Mononuclear phagocyte system
THA: Total hip arthroplasty
PICOS: Patient, intervention, comparison, outcomes, study design model
BMI: Body mass index
KSS: Knee Society Score
CI: Confidence interval
IRR: Incidence rate ratio
SMD: Standardised mean difference
RTKA: Revision total knee arthroplasty
WOMAC: Western Ontario and McMaster Universities Osteoarthritis Index
ROM: Range of motion
UCLA: University of California Los Angeles
LEAS: Lower extremity activity score
CR: Cruciate-retaining
PS: Posterior stabilised
